# Associations between Kidney Disease Progression and Metabolomic Profiling in Stable Kidney Transplant Recipients—A 3 Year Follow-Up Prospective Study

**DOI:** 10.3390/jcm13195983

**Published:** 2024-10-08

**Authors:** Titus Andrian, Lucian Siriteanu, Luminița Voroneanu, Alina Nicolescu, Calin Deleanu, Andreea Covic, Adrian Covic

**Affiliations:** 1 Department of Internal Medicine, University of Medicine and Pharmacy “Grigore T. Popa”, 700115 Iași, Romania; titus.andrian@umfiasi.ro (T.A.); luminita.voroneanu@umfiasi.ro (L.V.); andreea.covic@umfiasi.ro (A.C.); adrian.covic@umfiasi.ro (A.C.); 2 Nephrology, Dialysis and Transplantation Clinic, Clinical Hospital “Dr. C. I. Parhon”, 700503 Iași, Romania; 3 “Costin D. Nenitescu” Institute of Organic and Supramolecular Chemistry, 060023 Bucharest, Romania; alina220382@yahoo.com (A.N.); calin.deleanu@yahoo.com (C.D.); 4 “Petru Poni” Institute of Macromolecular Chemistry, 700487 Iași, Romania

**Keywords:** metabolomic, nuclear magnetic resonance, kidney transplant, allograft failure, glomerular filtration rate slope

## Abstract

**Background:** kidney transplant recipients are exposed to multiple pathogenic pathways that may alter short and long-term allograft survival. Metabolomic profiling is useful for detecting potential biomarkers of kidney disease with a predictive capacity. This field is still under development in kidney transplantation and metabolome analysis is faced with analytical challenges. We performed a cross-sectional study including stable kidney transplant patients and aimed to search for relevant associations between baseline plasmatic and urinary metabolites and relevant outcomes over a follow-up period of 3 years. **Methods:** we performed a cross-sectional study including 72 stable kidney transplant patients with stored plasmatic and urinary samples at the baseline evaluation which were there analyzed by nuclear magnetic resonance in order to quantify and describe metabolites. We performed a 3-year follow-up and searched for relevant associations between renal failure outcomes and baseline metabolites. Between-group comparisons were made after classification by observed estimated glomerular filtration rate slope during the follow-up: positive slope and negative slope. **Results**: The mean estimated GFR (glomerular filtration rate) was higher at baseline in the patients who exhibited a negative slope during the follow-up (63.4 mL/min/1.73 m^2^ vs. 55.8 mL/min/1.73 m^2^, *p* = 0,019). After log transformation and division by urinary creatinine, urinary dimethylamine (3.63 vs. 3.16, *p* = 0.027), hippuric acid (7.33 vs. 6.29, *p* = 0.041), and acetone (1.88 vs. 1, *p* = 0.023) exhibited higher concentrations in patients with a negative GFR slope when compared to patients with a positive GFR slope. By computing a linear regression, a significant low-strength regression equation between the log 2 transformed plasmatic level of glycine and the estimated glomerular filtration rate was found (*F* (1,70) = 5.15, *p* = 0.026), with an *R*^2^ of 0.069. Several metabolites were correlated positively with hand grip strength (plasmatic tyrosine with r = 0.336 and *p* = 0.005 and plasmatic leucine with r = 0.371 and *p* = 0.002). Other urinary metabolites were found to be correlated negatively with hand grip strength (dimethylamine with r = −0.250 and *p* = 0.04, citric acid with r = −0.296 and *p* = 0.014, formic acid with r = −0.349 and *p* = 0.004, and glycine with r = −0.306 and *p* = 0.01). **Conclusions**: some metabolites had different concentrations compared to kidney transplant patients with negative and positive slopes, and significant correlations were found between hand grip strength and urinary and plasmatic metabolites.

## 1. Introduction

Kidney transplantation has been proven to be a beneficial procedure for waitlisted end-stage kidney disease patients with a suitable profile. Both graft and patient survival rates have improved over time, especially in the short term. In order to significantly impact the long-term survival of kidney transplant recipients, there is a need for a holistic approach to assess, prevent, and treat the factors that may alter the graft health [1]. Recent efforts to predict the risk of allograft failure have resulted in validated multimodal tools that aggregate clinical, biological, immunologic, and histologic parameters [2,3]. Biomarker research in the area of kidney transplantation is fundamental, and the urgent need for the discovery of robust and powerful predictive tools remains relevant [4]. Metabolomic profiling is an increasingly used tool to attain better characterization of pathogenic mechanisms in kidney disease. Recent large cohort studies have shown significant associations between plasmatic and urinary metabolites and kidney failure or even death in chronic kidney disease (CKD) patients [5]. Certain serum or urinary metabolites have the potential to improve prognostic assessments [6]. Here, we perform a metabolomic analysis of plasma and urine of stable kidney transplant recipients and analyze differences among patients with different renal function trajectories based on estimation of the glomerular filtration rate slope. We also seek relevant associations between clinical and biological factors that impact kidney allograft survival and profiled metabolites.

## 2. Methods

The study was approved by the Ethics Committee of both the University of Medicine and Pharmacy “Gr. T. Popa” and the “Dr. C. I. Parhon” Clinical Hospital (approval code 179 from 5 May 2021). The study was conducted according to the guidelines of the Declaration of Helsinki. Informed consent of inclusion and of biological sampling was obtained from all subjects included in the study. We included non-diabetic, adult patients with a stable renal function, no recent hospitalizations, and no significant comorbidities (absence of end-stage liver disease, cancer, or congestive heart failure).

At the moment of inclusion, patients underwent a detailed evaluation comprising an ambulatory blood pressure measurement, routine biochemistry, echocardiography, arterial stiffness by measurement of carotid–femoral pulse wave velocity (SphygmoCor^®^), hand grip strength, and body composition assessment by bioimpedance (BCM-Body Composition Monitor^®^, Fresenius Medical Care, St. Wendel, Germany). Centrifugated biological samples (plasma and urine) for metabolomic studies were collected once for all patients at evaluation (baseline) and were stored at −80 °C for one year.

Using nuclear magnetic resonance (NMR), we profiled and quantified metabolites. One hour before NMR analysis, the serum samples were allowed to thaw at room temperature. The NMR samples were prepared as 1:1 blood serum to phosphate buffer solutions, gently homogenized in a plasma rotator and subsequently transferred in 5 mm NMR tubes (Wilmad 507). The Na_2_HPO_4_ buffer in D_2_O also contained 5 mM sodium 3-(trimethylsilyl)-[2,2,3,3-d_4_]-1-propionate (TSP) and NaN_3_. The NMR experiments were performed at 600 MHz using a Bruker Avance III HD instrument using an inverse detection NMR probe with gradients on the *z*-axis. Five different NMR experiments were performed at 310.0 K, using SOPs delivered through Bruker Biospin IVDr methods V.1.0. The experiments performed included the ^1^H NMR spectrum, the J-resolved 2D spectrum, the 1D diffusion filtered spectrum, the CPMG spectrum, and ^1^H gradient profile. An investigation into 12 urinary metabolites (creatinine, dimethylamine, alanine, glycine, proline betaine, valine, hippuric acid, citric acid, formic acid, succinic acid, acetoacetic acid, acetone, and D-glucose) was performed by NMR under similar conditions. Thus, a 9:1 urine to buffer was used and the same types of NMR spectra were recorded at 300.0 K. For this type of studies, the buffer was based on KH_2_PO_4_ and the mixing of constituents was performed with vigorous shaking in a vortex shaker. Urinary metabolites were reported as a ratio by dividing them by urinary creatinine. Estimated glomerular filtration rate (eGFR) was reported based on creatinine and calculated with the recently validated race-free kidney recipient specific equation [7]. Patients were followed-up through routinely scheduled ambulatory visits for a period of three years.

For statistical analysis, missing values were assumed to be below the lower limit of detection and these values were imputed (minimum values divided by 5). Metabolite levels were all log 2 transformed in order to reduce skewness. Demographic, clinical, and laboratory characteristics were reported using mean (± standard deviation) or counts (n) and fractions (%). Differences among groups were assessed using the independent samples *t*-test, the Chi-square test, or the Mann–Whitney U test as appropriate, depending on the type of variables. Potentially relevant associations (with glomerular filtration rate, proteinuria, or glomerular filtration rate slope) were verified by performing a linear regression, regressing on log-transformed metabolites. The slope was estimated using the least squares linear regression of all glomerular filtration rates measurements on time with several time points (baseline, 6 months, 12 months, 24 months, 36 months) [8]. The strength of the relationships between two variables was assessed by calculating the correlation coefficients (Pearson’s or Spearman, depending on the type of variables). We also examined the associations between metabolites and time to kidney failure (defined by any of the following: progression to stage 5 of CKD, 40% loss of GFR from baseline, need for dialysis, re-transplantation, or death) by using Cox proportional hazard regression model and reported the hazard ratios and the corresponding 95% confidence intervals and *p*-values. Statistical analysis was performed using R version 4.2.1 in RStudio (https://cran.r-project.org) accessed on 15 May 2024 and IBM SPSS Statistics 25.

## 3. Results

Baseline characteristics of the 72 included patients are presented in Table 1. Patients were divided into two groups depending on the value of the GFR slope. Participants with a negative slope had a higher GFR at baseline, with no other significant difference (Table 1).

**Figure 1:**
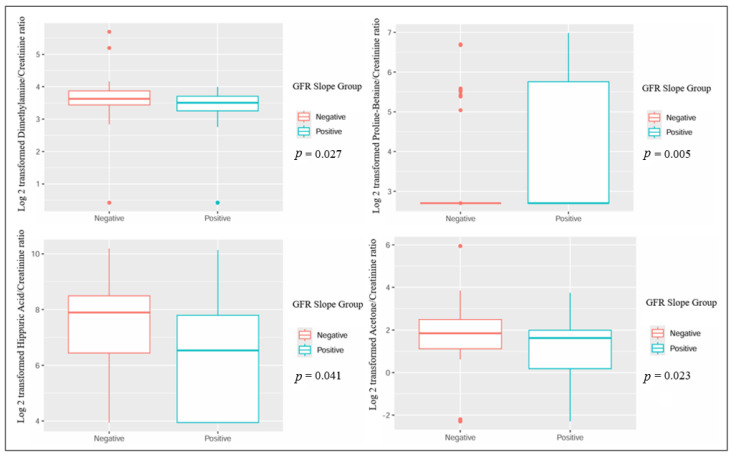
Significant differences in urinary metabolites in kidney transplant recipients with different GFR slope trajectories.

**Figure 2:**
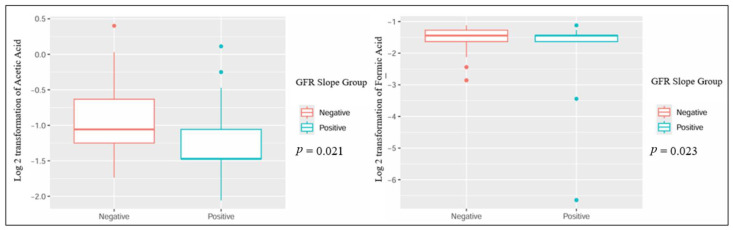
Significant differences in plasmatic metabolites in kidney transplant recipients with different GFR slope trajectories.

When comparing patients based on the GFR slope, several metabolites showed statistically significant differences: urinary dimethylamine, hippuric acid, and acetone exhibited higher concentrations in patients with a negative GFR slope. Conversely, urinary proline betaine was higher in patients with a positive GFR slope (Table 2 and Figure 1). Also, the plasmatic concentration of acetic acid was higher in patients with a positive slope, while formic acid had lower values when compared to patients with a negative slope (Table 3 and Figure 2).

**Figure 3:**
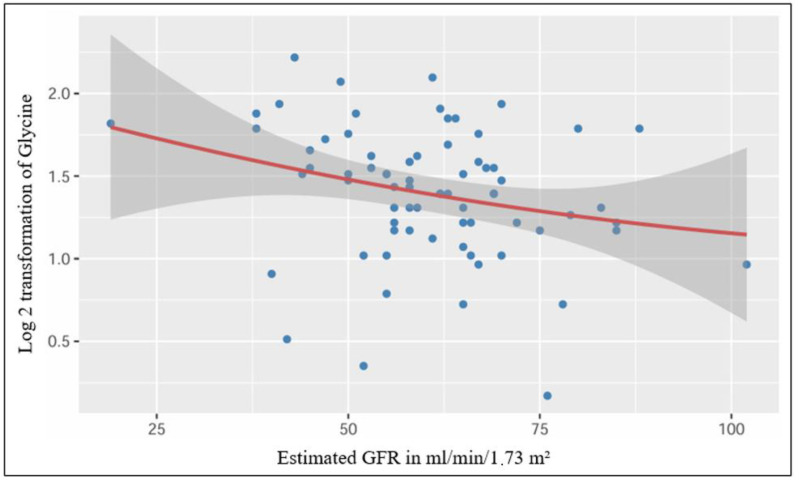
Significant linear relationship between plasmatic Glycine and estimated GFR.

After performing a simple linear regression, we found a significant low-strength regression equation between the log 2 transformed plasmatic level of glycine and the estimated glomerular filtration rate (*F* (1,70) = 5.15, *p* = 0.026), with an *R*^2^ of 0.069 (Figure 3).

We found no association between metabolite levels and the renal outcome in the univariate Cox models. At the end of follow-up there were no significant differences between groups regarding renal failure outcomes and clinical adverse events (Table 4).

**Figure 4:**
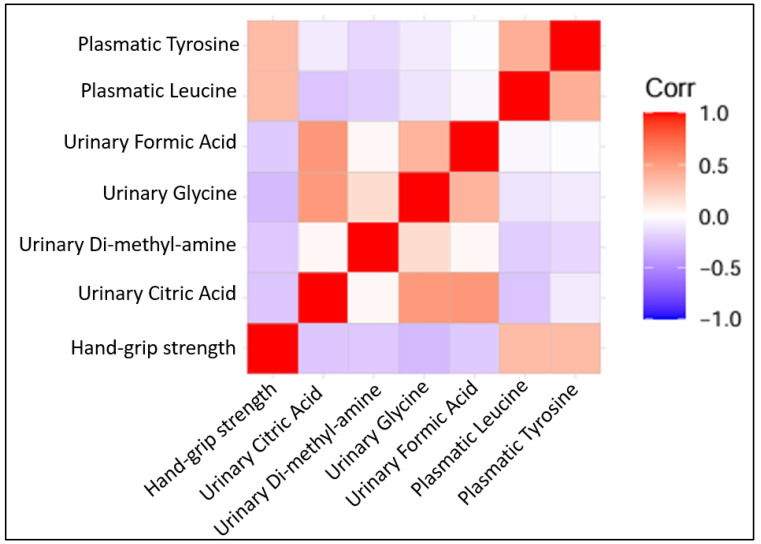
Correlation plots of statistically significant Pearson’s correlation among quantified metabolites (all log 2 transformed) with each other and with hand grip strength.

Several metabolites were correlated positively with hand grip strength (plasmatic tyrosine with r = 0.336 and *p* = 0.005, plasmatic leucine with r = 0.371 and *p* = 0.002). Other urinary metabolites were found to be correlated negatively with hand grip strength (dimethylamine with r = −0.250 and *p* = 0.04, citric acid with r = −0.296 and *p* = 0.014, formic acid with r = −0.349 and *p* = 0.004, glycine with r = −0.306 and *p* = 0.01) (Figure 4).

## 4. Discussion

Our study found statistically significant differences in metabolite concentrations between patients with a negative GFR slope when performing comparisons with patients that showed a positive GFR slope in the three-year follow-up study. Despite this, none of these metabolites were associated with CKD progression in our cohort of stable kidney transplant patients. This paradoxical finding has been previously reported and some authors have stated that the interpretation of the associations warrants attention because of possible nonspecific accumulations of metabolites in patients with declining renal function [6,9]. Consistent with our findings, other studies have also reported alterations to the metabolomic profiling in patients with different degrees of graft dysfunction [10,11].

Metabolomic studies have been developed with respect to CKD, and, specifically, kidney transplantation, in order to find biomarkers that possess diagnostic and prognostic information. A recent cross-sectional evaluation of a large population of CKD patients has found a modest improvement in risk prediction capability when incorporating serum or urinary metabolites [12]. Our study did not prove associations between the studied metabolites and hard endpoints of kidney failure in transplant recipients, adding to the body of evidence that, for the moment, suggests that allograft failure is best predicted by combining the estimated GFR, proteinuria, immunological, and histopathological parameters [2,13].

There has been a recent growth in the scientific literature addressing the metabolomic profile associated with kidney transplantation. The ability to fully characterize the changes in metabolites in complex medical cases, such as patients with different stages of allograft dysfunction and undergoing different immunosuppressive therapies, is challenging to attain, but it will provide useful insights with respect to interpreting different disease pathways [14]. Our study adds to this growing body of evidence and, also, brings novelty by describing associations with muscular metabolism and function. To the best of our knowledge, our study is the first one to describe correlations between metabolites and hand grip strength in patients who have undergone kidney transplantation. Low muscle strength has been proven to be common in kidney transplant recipients and is associated to a poor prognosis, low quality of life, or malnutrition [15,16,17]. Leucine, which we found to be positively correlated with hand grip strength, is an essential amino acid obtained through food. Oxidation occurs in the kidneys and in the muscles, revealing the close relationship with muscular health [18].

Our study reports a significant linear relationship between plasmatic glycine and estimated GFR. A recent study has synergistically studied the genomic and metabolomic characteristics that impact CKD progression, revealing that mannose and glycine are metabolites that significantly correlate with kidney disease [19]. However, glycine is a biomarker related to creatine metabolism and associations with GFR estimated on the basis of creatinine may stem from this relationship [20].

Our work presents several limitations: evidently, the sample size may have prevented us from obtaining other significant results. Also, external validation was not performed in other cohorts. The methodology used for quantification based on nuclear magnetic resonance presents limited sensitivity when compared to mass-spectrometry-based approaches. Given that we performed only a one-time baseline evaluation of the metabolome at the patient inclusion stage, this limited our capacity to provide further insights into the possible time-dependent changes in kidney transplanted patients.

We believe that our study also presents notable strengths. We enrolled stable kidney transplant recipients, representing a population with a poorly characterized metabolomic profile given the many possible interactions modulated by immunosuppressive therapy or gut dysbiosis. Assessing metabolites from both urine and plasma enabled a more comprehensive overview of the associations with clinical outcomes. We found relevant associations with different metabolic pathways and performed an extensive evaluation of the included patients at the time of enrollment. Also, the prospective nature of the research allowed us to evaluate the relationship between kidney function dynamic changes and baseline metabolomic profiling. Moreover, the patients included in our study were not diabetic, allowing us to examine the metabolomic profile irrespective of significant metabolic derangements. Diabetic patients have been shown to exhibit different patterns of metabolomic signatures when compared to patients with glomerular disease [21,22].

Metabolome studies are a very active subject of research, and new metabolites are continuously found to exhibit associations with outcomes in nephrology [5]. Further studies will help understand the utility of metabolites as biomarkers.

Taken together, our findings highlight that transplant recipients with distinct trajectories of kidney function during a follow-up of three years exhibit a slightly different plasmatic and urinary metabolomic profile. Also, plasmatic glycine was the only metabolite significantly correlated with the estimated glomerular filtration rate, and we found multiple significant associations between several urinary and plasmatic metabolites and hand grip strength. Our study offers new insights into the complex characterization of metabolic pathways that mediate kidney disease in renal transplant recipients.

## Figures and Tables

**Table 1:** Baseline characteristics of included patients.

Parameter	Negative GFR Slope, *n* = 45	Positive GFR Slope, *n* = 27	*p*-Value
Women, ***n*** (%)	15 (33.3%)	10 (37%)	0.749
Age, mean (±standard deviation)	46.3 (±11.8)	45.2 (±11.7)	0.716
Years since transplantation, mean (±standard deviation)	7.4 (±6.4)	6 (±4.8)	0.33
CKD etiology Glomerulonephritis, ***n*** (%)	27 (60%)	13 (48.1%)	0.614
Deceased donor, ***n*** (%)	23 (51.1%)	14 (51.9%)	0.951
Preemptive transplantation, ***n*** (%)	13 (29.5%)	7 (25.9%)	0.631
Dialysis vintage, mean (±standard deviation)	2.16 (±2.8)	1.8 (±1.9)	0.576
Estimated GFR in mL/min/1.73 m^2^, mean (±standard deviation)	63.4 (±13.9)	55.8 (±11.1)	0.019
GFR slope in mL/min/1.73 m^2^, mean (±standard deviation)	−3.99 (±4.1)	1.89 (±1.7)	<0.001
Smokers, ***n*** (%)	8 (17.8%)	5 (18.5%)	0.937
Systolic blood pressure in mmHg, mean (±standard deviation)	140.8 (±15.7)	142.2 (±18)	0.731
Body mass index in kg/m^2^, mean (±standard deviation)	26.2 (± 3.9)	26.3 (±4.4)	0.866
Hemoglobin in g/dL, mean (±standard deviation)	13.5 (±1.5)	13.7 (±1.4)	0.586
Urinary protein on creatinine ratio in mg/g, mean (±standard deviation)	0.43 (±1.07)	0.21 (±0.58)	0.332
Total cholesterol in mg/dL, mean (±standard deviation)	200 (±47.5)	214.6 (±40.7)	0.203
Triglycerides in mg/dL, mean (±standard deviation)	154.2 (±71.6)	156.7 (±83)	0.895
Tacrolimus-based immunosuppression, ***n*** (%)	26 (57.8%)	19 (70.4%)	0.467
Mycophenolate use, ***n*** (%)	44 (97.8%)	26 (96.3%)	0.711
Corticosteroid use, ***n*** (%)	32 (71.1%)	21 (77.8%)	0.534
Pulse wave velocity in cm/s, mean (±standard deviation)	6.47 (±1.83)	6.47 (±1.44)	0.992
Hand grip strength in kg, mean (±standard deviation)	34.6 (±8.1)	38.3 (±10.5)	0.102

**Table 2:** Urinary metabolites at baseline.

Metabolite	Negative GFR Slope, *n* = 45	Positive GFR Slope, *n* = 27	*p* Value
Log 2 transformation of dimethylamine/creatinine ratio, mean (±standard deviation)	3.63 (±0.69)	3.16 (±1.04)	0.027
Log 2 transformation of alanine/creatinine ratio, mean (±standard deviation)	3.56 (±1.39)	3.40 (±1.62)	0.680
Log 2 transformation of glycine/creatinine ratio, mean (±standard deviation)	4.42 (±1.61)	4.09 (±1.83)	0.439
Log 2 transformation of proline betaine/creatinine ratio, mean (±standard deviation)	3.18 (±1.15)	4.14 (±1.62)	0.005
Log 2 transformation of valine/creatinine ratio, mean (±standard deviation)	0.68 (±1.56)	0.66 (±1.78)	0.960
Log 2 transformation of hippuric acid/creatinine ratio, mean (±standard deviation)	7.33 (±1.93)	6.29 (±2.12)	0.041
Log 2 transformation of citric acid/creatinine ratio, mean (±standard deviation)	5.83 (±1.91)	5.65 (±1.87)	0.699
Log 2 transformation of formic acid/creatinine ratio, mean (±standard deviation)	1.23 (±1.8)	1.02 (±1.51)	0.611
Log 2 transformation of succinic acid/creatinine ratio, mean (±standard deviation)	2.83 (±1.44)	2.91 (±1.22)	0.815
Log 2 transformation of acetoacetic acid/creatinine ratio, mean (±standard deviation)	3.71 (±1.42)	3.41 (±1.52)	0.408
Log 2 transformation of acetone/creatinine ratio, mean (±standard deviation)	1.88 (±1.33)	1 (±1.82)	0.023

**Table 3:** Plasmatic metabolites at baseline.

Metabolite	Negative GFR Slope, *n* = 45	Positive GFR Slope, *n* = 27	*p* Value
Log 2 transformation of alanine, mean (±standard deviation)	2.11 (±0.29)	2.14 (±0.21)	0.587
Log 2 transformation of glutamic acid, mean (±standard deviation)	2.16 (±0.5)	2.03 (±0.49)	0.275
Log 2 transformation of glutamine, mean (±standard deviation)	2.77 (±0.69)	2.87 (±0.34)	0.509
Log 2 transformation of glycine, mean (±standard deviation)	1.43 (±0.37)	1.33 (±0.43)	0.284
Log 2 transformation of histidine, mean (±standard deviation)	0.82 (±0.3)	0.77 (±0.31)	0.506
Log 2 transformation of isoleucine, mean (±standard deviation)	−0.13 (±0.36)	−0.19 (±0.38)	0.523
Log 2 transformation of leucine, mean (±standard deviation)	0.76 (±0.34)	0.74(±0.33)	0.790
Log 2 transformation of methionine, mean (±standard deviation)	−1.14 (±1.48)	−1.30 (±1.52)	0.661
Log 2 transformation of phenylalanine, mean (±standard deviation)	0.79 (± 0.34)	0.64 (±0.3)	0.08
Log 2 transformation of tyrosine, mean (±standard deviation)	0.08 (±0.3)	0.13 (±0.35)	0.553
Log 2 transformation of valine, mean (±standard deviation)	1.59 (±0.23)	1.57 (±0.25)	0.694
Log 2 transformation of acetic acid, mean (±standard deviation)	−0.95 (±0.46)	1.57 (±0.25)	0.021
Log 2 transformation of formic acid, mean (±standard deviation)	−1.53 (±0.43)	−2.14 (±1.67)	0.023
Log 2 transformation of lactic acid, mean (±standard deviation)	4.71 (±0.49)	4.69 (±0.56)	0.868
Log 2 transformation of acetone, mean (±standard deviation)	−1.87 (±1.07)	−2.05 (±1.2)	0.513

**Table 4:** Patient characteristics at the end of the follow-up.

Parameter	Negative GFR Slope, *n* = 41	Positive GFR Slope, *n* = 26	*p*-Value
Estimated GFR in mL/min/1.73 m^2^, mean (±standard deviation)	53.5 (±17)	60.6 (±11.5)	0.058
Hemoglobin in g/dL, mean (±standard deviation)	12.1 (±2.2)	13.4 (±1.3)	0.01
Urinary protein in creatinine ratio in mg/g, mean (±standard deviation)	0.57 (±1.07)	0.25 (±0.57)	0.177
Patients with infection episodes, ***n*** (%)	17 (38.6%)	11 (40.7%)	0.860
Patients with rejection episodes, ***n*** (%)	9 (20.5%)	5 (18.5%)	0.842
Renal outcomes (one of progression to end-stage kidney disease, dialysis, or retransplantation, 40% loss of GFR, death)	6 (13.6%)	1 (3.7%)	0.173

## Data Availability

Data can be provided upon request.
